# CATS (FAM64A) abnormal expression reduces clonogenicity of hematopoietic cells

**DOI:** 10.18632/oncotarget.11724

**Published:** 2016-08-31

**Authors:** Isabella Barbutti, Juliana M. Xavier, João Agostinho Machado-Neto, Lauremilia Ricon, Fabiola Traina, Stefan K. Bohlander, Sara Teresinha Olalla Saad, Leticia Fröhlich Archangelo

**Affiliations:** ^1^ Hematology and Hemotherapy Center, State University of Campinas (UNICAMP), Carlos Chagas 480, Campinas-SP, Brazil; ^2^ Department of Internal Medicine, Ribeirão Preto Medical School, University of São Paulo, Ribeirão Preto, São Paulo, Brazil; ^3^ Department of Molecular Medicine and Pathology, The University of Auckland, Auckland, New Zealand; ^4^ Department of Cellular and Molecular Biology and Pathogenic Bioagents, Ribeirão Preto Medical School, University of São Paulo, Ribeirão Preto, São Paulo, Brazil

**Keywords:** CATS (FAM64A), proliferation, clonogenicity, CALM/AF10, leukemogenesis

## Abstract

The CATS (FAM64A) protein interacts with CALM (PICALM) and the leukemic fusion protein CALM/AF10. CATS is highly expressed in leukemia, lymphoma and tumor cell lines and its protein levels strongly correlates with cellular proliferation in both malignant and normal cells. In order to obtain further insight into CATS function we performed an extensive analysis of CATS expression during differentiation of leukemia cell lines. While CATS expression decreased during erythroid, megakaryocytic and monocytic differentiation, a markedly increase was observed in the ATRA induced granulocytic differentiation. Lentivirus mediated silencing of CATS in U937 cell line resulted in somewhat reduced proliferation, altered cell cycle progression and lower migratory ability *in vitro;* however was not sufficient to inhibit tumor growth in xenotransplant model. Of note, CATS knockdown resulted in reduced clonogenicity of CATS-silenced cells and reduced expression of the self-renewal gene, *GLI-1*. Moreover, retroviral mediated overexpression of the murine Cats in primary bone marrow cells lead to decreased colony formation. Although our *in vitro* data suggests that CATS play a role in cellular processes important for tumorigenesis, such as cell cycle control and clonogenicity, these effects were not observed *in vivo*.

## INTRODUCTION

The t(10;11)(p13;q14) translocation leads to the fusion of the *CALM* (*PICALM*) and *AF10* genes [1]. *CALM/AF10* fusions are observed in acute myeloid leukemia (AML), acute lymphoblastic leukemia (ALL) and in lymphoma [2–5], being very frequent in gamma/delta lineage T-acute lymphoblastic leukemias [6–8].

The expression of CALM/AF10 leads to the development of leukemia in murine bone marrow transplantation and transgenic models [9–12], and increasing evidence suggests that CALM/AF10 exerts its leukemogenic potential through transcriptional deregulation of target genes, including the HOXA gene cluster, therefore interfering with normal hematopoietic differentiation [9, 13–15], through increased genomic instability by reducing global histone H3K79 methylation [16, 17] and through a novel proposed mechanism mediated by the CRM1-dependent nuclear export pathway [18]. Identification of several CALM/AF10 interacting proteins (*e.g*. CATS (FAM64A), DOTL1, IKAROS, FHL2), has shed light on the molecular mechanism relevant for CALM/AF10-mediated leukemogenesis and the possible involvement of these proteins in malignant transformation [15, 19–22].

CATS (FAM64A; Gene ID: 54478) was initially identified as a CALM interacting partner [19]. The findings that expression of CATS (FAM64A) markedly increased the nuclear localization of CALM/AF10 [19] and that the murine *Cats (Fam64A)* transcripts were up-regulated in hematopoietic cells (B220^+^ lymphoid cells) transformed by CALM/AF10 in comparison to the same subpopulation from non-leukemic mice [10, 23], suggested that CATS (FAM64A) may play a role in CALM/AF10-mediated transformation. In agreement with that, CATS (FAM64A) functions as a transcriptional repressor [19] capable of antagonizing the transactivation activity of the leukemic fusion protein CALM/AF10 in a GAL4-based transactivation assay [24]. However, whether CATS (FAM64A) contributes to leukemogenesis remains to be determined.

In normal adult tissue, *CATS (FAM64A)* is predominantly expressed in the lymphoid compartment, whereas it is highly expressed in leukemia, lymphoma and tumor cell lines. The protein level of CATS (FAM64A) strongly correlates with cellular proliferation in both normal and malignant cells [23].

Zhao and co-workers reported that CATS (FAM64A) (referred to as RCS1 in their study), is a mitotic regulator that controls the metaphase-to-anaphase transition [25]. Additional roles for CATS as a neuronal protein that is co-expressed and interacts with the cellular prion protein (PrPC) have also been proposed [26, 27]. Most recently, CATS (FAM64A) was found among the three most upregulated genes, whose high expression is associated with poor prognosis of more aggressive triple-negative breast cancer (TNBC) [28].

In order to gain further insight into CATS function we performed an extensive analysis of CATS expression during differentiation of leukemia cell lines and investigated the effect of CATS silencing in the CALM/AF10-positive U937 leukemia cell line as well as the effect of Cats overexpression in murine primary bone marrow cells. Here we show that changes in CATS expression affect cell proliferation, cell cycle control and clonogenicity of hematopoietic cells.

## RESULTS

### CATS expression decreases during induced differentiation of leukemia cell lines

**Figure 1:**
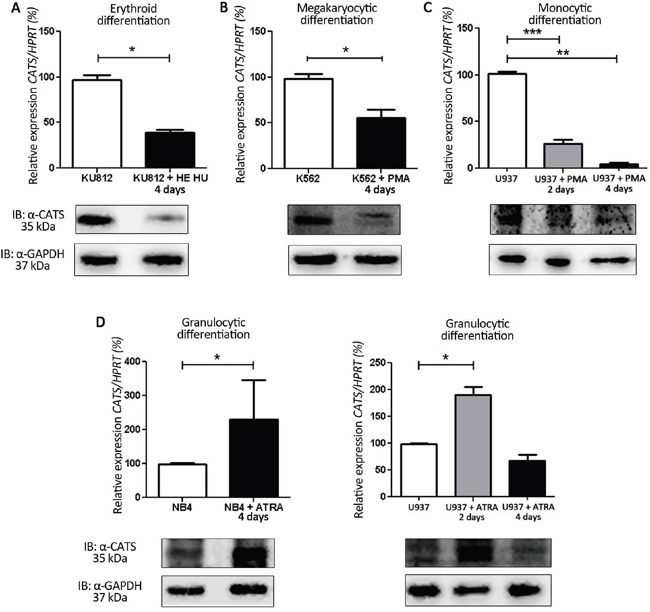
CATS expression during induced differentiation of leukemia cell lines Relative *CATS* expression at days 2 and/or 4 of differentiation. Upper panels: *CATS* mRNA levels normalized by *HPRT*. Lower panels: Representative Western blots of total cell extracts. Membrane was blotted against anti-CATS 2C4 and anti-GAPDH, used as a loading control. **A.** HE + HU induced erythroid differentiation of KU812 cells. Results are shown as mean ± SD of three independent experiments. **B.** PMA induced megakaryocytic differentiation of K562 cells. Results are shown as mean ± SD of three independent experiments. **C.** PMA induced monocytic differentiation of U937 cells. Results are shown as mean ± SD of four independent experiments. **D.** ATRA induced granulocytic differentiation of NB4 (left panel) and U937 cells (right panel). Results are shown as mean ± SD of six and four independent experiments, respectively. *p<0.05; **p<0.01 and ***p<0.001, Student's t test.

We first investigated CATS gene and protein expression during induced differentiation of leukemia cell lines into erythrocytes, megakaryocytes, monocytes and granulocytes (Figure 1 and Supplementary Figure S1). *CATS* expression decreased during erythroid (by 60%), megakaryocytic (by 43%) and monocytic (by 65% at day 2, and by 96% at day 4) differentiation (Figure 1A-1C). However, *CATS* expression increased by 2 fold during granulocytic differentiation of both NB4 (at day 4) and U937 cells (at day 2) (Figure 1D). At day 4 of U937 granulocytic differentiation, CATS expression returned to its initial level. Expression of CATS protein followed the same pattern as its transcript levels (Figure 1D, lower panels).

### CATS knockdown reduces cell proliferation of the U937 cell line *in vitro*

U937 cells were transduced with lentiviral particles expressing a pool of multiple shRNAs for silencing CATS. This approach provides a selective reduction of off-target effects since the pool silence the intended target but have unique off-target signatures [29]. CATS silencing was efficiently achieved at both mRNA and protein levels in shCATS transduced cells when compared to shControl cells (Supplementary Figure S2A).

**Figure 2:**
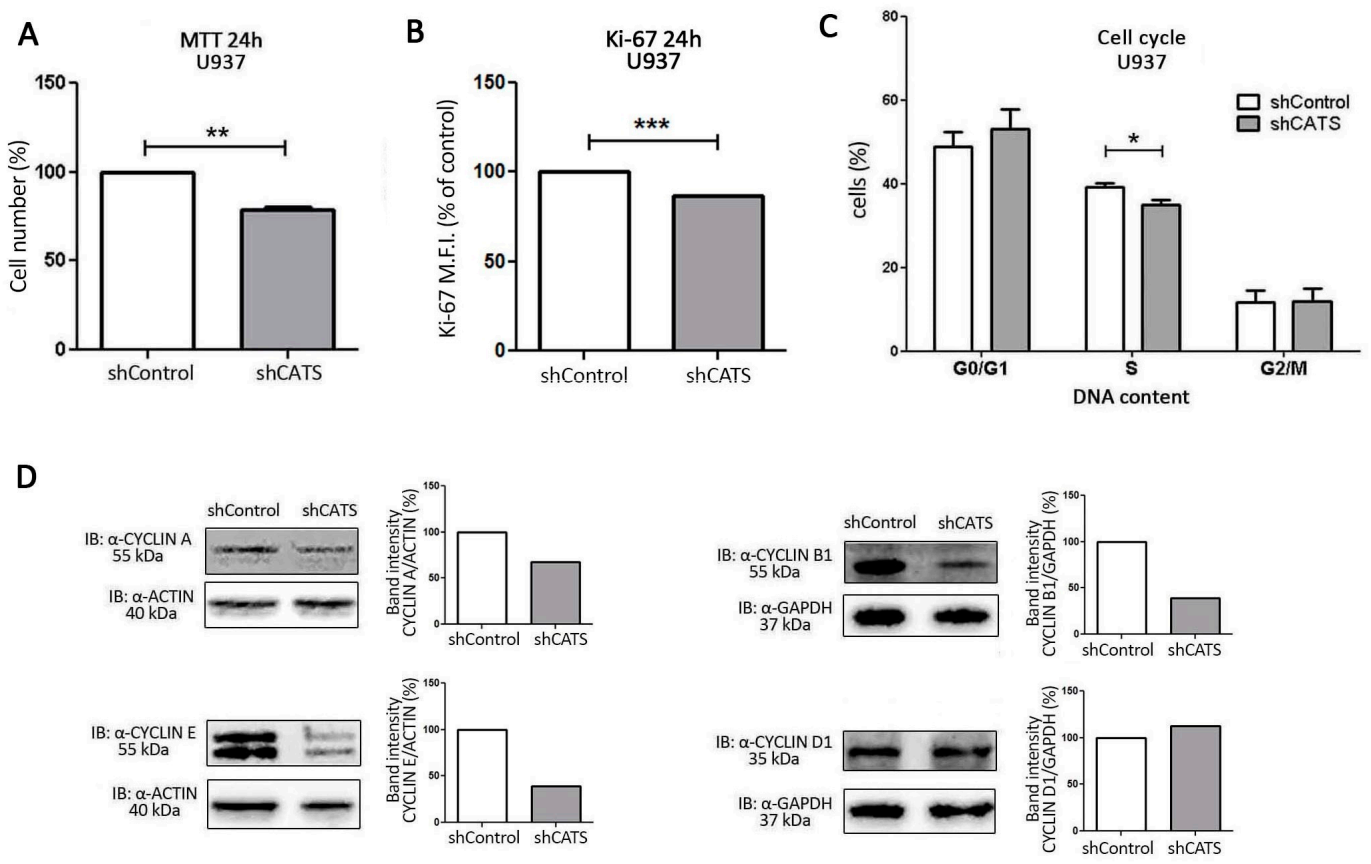
CATS knockdown reduces proliferation of U937 cells *in vitro* Cell proliferation was determined **A.** by MTT assay after 24h of normal culture conditions. Results are shown as mean ± SD of three independent sixplicates experiments. **B.** Ki-67 mean of fluorescence intensity (M.F.I.) determined by flow cytometry after 24h incubation of shCATS and normalized by shControl cells. Results are shown as mean ± SD of three independent experiments. **C.** Percentage of cells on different cell cycle phases determined by flow cytometry. Results are shown as mean ± SD of three independent triplicate experiments. **D.** Western blot analysis of shControl and shCATS total cell extracts. Membrane was blotted against anti-CYCLIN A, anti-CYCLIN E, anti-CYCLIN B1, anti-CYCLIN D1, and anti-ACTIN or anti-GAPDH, as a control for equal sample loading. The bar graphs represent densitometric analysis of blots relative to control set as 100% (UN-SCAN-IT software). *p<0.05; **p<0.01, ***p< 0.001, Student's t test.

Cell number decreased by approximately 20% upon CATS depletion at two different time points: 24h (Figure 2A) and 48h (Supplementary Figure S3A), as assessed by MTT. No difference in apoptosis was encountered (Supplementary Figure S3B), leading us to believe the difference in viability was due to reduced cell proliferation. Accordingly, Ki-67 analysis revealed reduced proliferation of CATS silenced cells by 13% (Figure 2B). As shown by cell cycle analysis, a lower percentage (12% lower) of cells in the S phase of the cell cycle was observed in shCATS cells in comparison to shControl cells (Figure 2C). These results indicate a reduced proliferation of U937 cells upon CATS depletion. Alteration in the cell cycle control was further demonstrated by decreased expression of CYCLIN A, E and B1 in shCATS cell lysates, as demonstrated by Western blotting. Levels of CYCLIN D1 remained unaltered in CATS knockdown cells when compared to control cells (Figure 2D).

### CATS knockdown does not interfere with growth of U937 cells in a xenotransplant model

**Figure 3:**
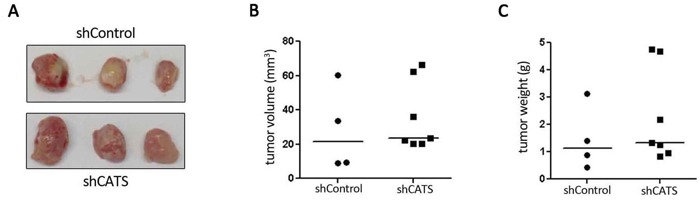
CATS knockdown do not interfere with tumor growth *in vivo* **A.** Tumors formed after inoculation of 1 × 10^7^ U937 shCATS or shControl cells subcutaneously into NOD/SCID mice and excised after 12 days of growth. **B.** Tumors volume. **C.** Tumors weight.

A xenotransplant model was used to assess whether CATS depletion would inhibit tumor growth *in vivo*. No difference was observed in volume or weight of shCATS and shControl tumors after 12 days of tumor growth (Figure 3). *CATS* knockdown was confirmed on excised tumors samples (Supplementary Figure S4).

### CATS knockdown does not alter apoptosis of the U937 cells

**Figure 4:**
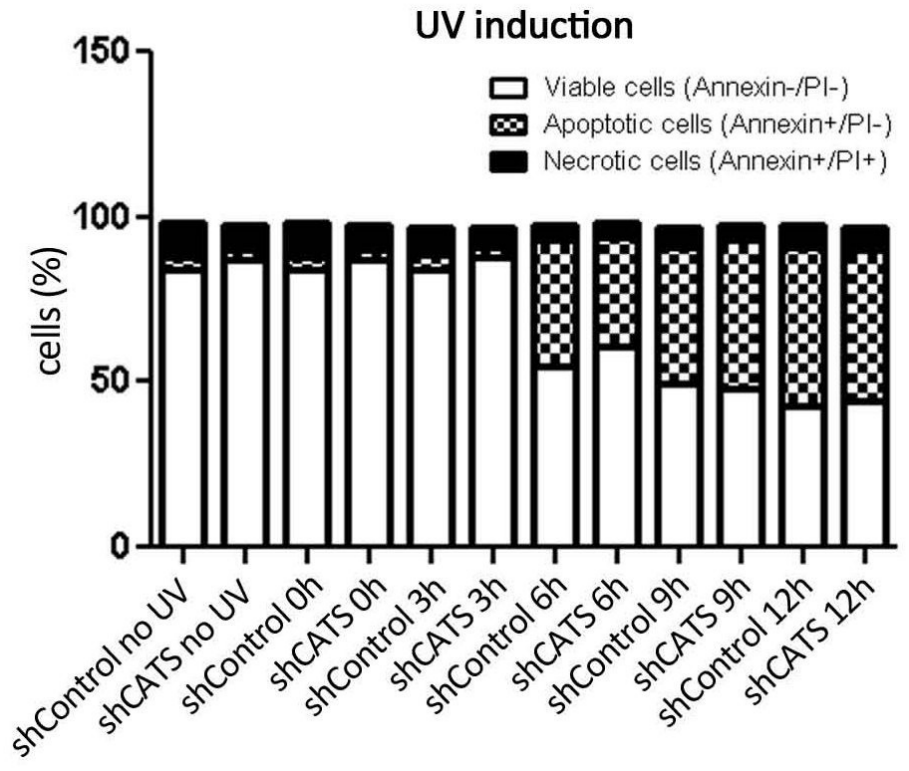
CATS knockdown does not alter cell death rate upon UV irradiation Apoptosis was determined by staining cells with Annexin V and PI, followed by flow cytometry analysis. Cells were submitted to UV irradiation (40 J/m^2^) and collected at the indicated time points (0, 3, 6, 9, and 12 hours). The data represents one experiment.

Recently, we identified CATS interaction with proteins involved in apoptotic response (unplublished data), therefore we wanted to investigate whether CATS plays a role in apoptosis. shCATS and shControl cells were induced to cell death upon UV irradiation exposition and assessed for annexin-V/PI staining. No difference in the percentage of viable versus apoptotic cells was observed (Figure 4).

### CATS depletion decreases migration of U937 cells

**Figure 5:**
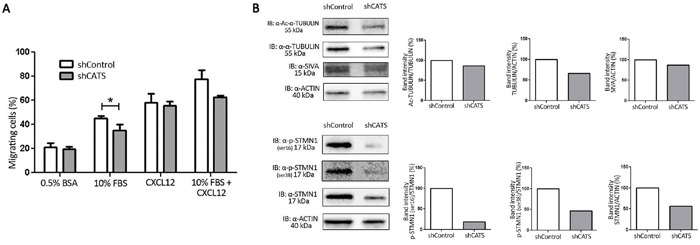
CATS knockdown reduced U937 cell migration towards serum containing media Migration was evaluated by counting the number of cells that passed through the 8μm pore-sized transwell plates and were present in the lower compartment after 24h exposure to the chemotatic stimuli. **A.** Migration towards 0.5% BSA (used as a control), 10% FBS, CXCL12 and 10% FBS + CXCL12 containing media. The bars represent the number of migrating cells normalized by the input, expressed as percentage. Results are shown as mean ± SD of at least three independent duplicate experiments. **B.** Western blot analysis of shControl and shCATS total cell extracts. Membrane was blotted against anti-Ac-α-TUBULIN, anti-α-TUBULIN, anti-SIVA and anti-ACTIN, used as a control for equal sample loading (upper panel) and against anti-STMN1 (Ser16), anti-STMN1 (Ser38), anti-STMN1 and anti-ACTIN, used as a control for equal sample loading (lower panel). The bar graphs represent densitometric analysis of blots relative to control set as 100% (UN-SCAN-IT software). *p<0.05, Student's t test.

We performed a transwell chemotaxis assay in order to investigate whether CATS contributes to cell migration. For that purpose we analyzed shCATS and shControl cell migration towards three different chemotatic stimuli: FBS (Fetal Bovine Serum), CXCL12 and FBS with CXCL12. Approximately 20% fewer shCATS cells than shControl cells migrated towards 10% FBS and 10% FBS with CXCL12, whereas migration towards CXCL12 was unaltered (Figure 5A). Cell migration towards 0.5% BSA (used as a control for the experiment) was low and equal for both shControl and shCATS.

Next, we investigated expression of proteins involved in microtubule dynamics and known to control cell migration. Western blot analysis revealed decreased expression of α-TUBULIN, and STATHMIN1, as well as reduced overall α-TUBULIN acetylation and STATHMIN1 phosphorylation on residues Ser16 and Ser38 (Figure 5B).

### CATS knockdown reduces clonogenicity of U937 cell line

**Figure 6:**
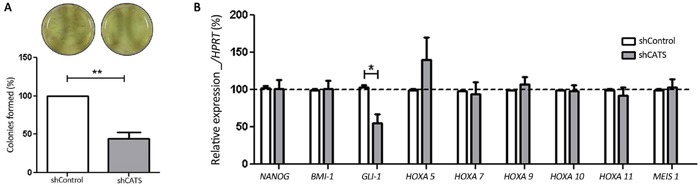
CATS knockdown reduces colony formation and *GLI-1* mRNA expression in U937 cells **A.** Colony formation assay. Colonies containing viable cells were stained with MTT after 8 days of culture. Images of representative culturing plates are shown. The bars represent the number of colonies formed normalized by the control and expressed as percentage. Results are shown as mean ± SD of six independent triplicates experiments. **B.** Relative expression of the self-renewal regulators (*NANOG*, *BMI-1* and *GLI-1*) and the CALM/AF10-leukemia deregulated genes (*HOXA5, HOXA7, HOXA9, HOXA10, HOXA11* and *MEIS1*) in shCATS and shControl cells. Results are shown as mean ± SD of at least three independent triplicates experiments. Expression levels of mRNA were normalized by *HPRT.* *p<0.05; **p<0.01, Student's t test.

We employed a colony formation assay to assess the clonogenic potential of the CATS depleted U937 cells on semisolid medium in the absence of growth factors. A significant decrease of 66% in colony number was observed for shCATS transduced U937 cells compared to the shControl transduced cells (Figure 6A).

In order to investigate a possible mechanism involved in the reduced ability of CATS depleted cells to form colonies, we analyzed the expression of the self-renewal related genes *NANOG* [30, 31], *BMI-1* [32] and *GLI-1* [33] in the shCATS and shControl cells. Interestingly, while expression of *NANOG* and *BMI-1* were not altered, a reduction of *GLI-1* expression ranging between 18% and 75% was observed in CATS depleted U937 cells (Figure 6B).

Since CATS interacts with the CALM/AF10 fusion protein which is present in the U937 cells, we sought to investigated whether CATS depletion in U937 cells would affect the expression of the CALM/AF10-leukemia deregulated genes *HOXA5, HOXA7, HOXA9, HOXA10, HOXA11* and *MEIS1*. Expression of *HOXA7, HOXA9, HOXA10, HOXA11*, and *MEIS1* genes were unaffected in U937 upon CATS depletion. Although not statistically significant, a tendency towards increased *HOXA5* expression was consistently observed in different samples of CATS-depleted U937 cells (Figure 6B). In CALM/AF10 leukemia, *HOXA5* overexpression results from CALM/AF10 recruitment of hDOT1L and local H3K79 hypermethylation at the *HOXA5* locus [15], whereas a global reduction of this epigenetic marker also occurs [16]. We analyzed global H3K79 methylation in shCATS transduced cells and shControl cells by Western blotting. The global H3K79 methylation levels in U937 cells were not affected by CATS depletion (Supplementary Figure S5).

### Cats overexpression reduces clonogenicity of mice primary bone marrow cells

**Figure 7:**
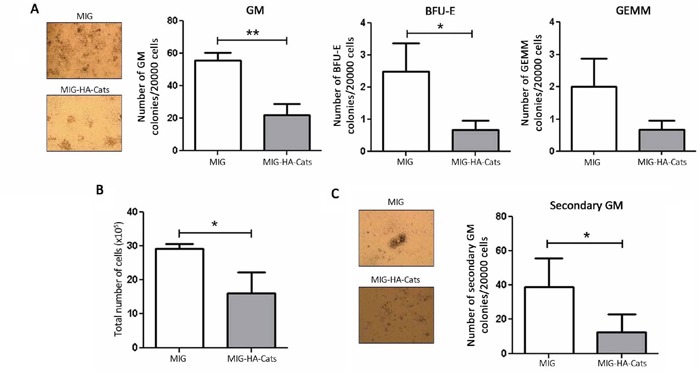
Cats overexpression reduces the number of colony formation and total cell number of primary bone marrow progenitor cells **A.** Transduced cells were cultured in myeloid-conditioned methylcellulose medium for 8 days after which colonies were counted. The bars represent the number of GM (granulocytes and monocytes), BFU-E (erythrocytes) and GEMM (progenitors) colonies and **B.** the total number of cells. **C.** Cells were replated in the same concentration and further cultured for 10 days, allowing secondary colony formation. The bars indicate the number of secondary GM colonies. Results are shown as mean ± SD of three independent experiments. *p<0.05; **p<0.01, Student's t test.

In order to investigate a possible role of Cats in the clonogenicity and/or differentiation potential of primary bone marrow cells, we retrovirally transduced murine progenitor cells to overexpress Cats (Supplementary Figure S2B) and performed colony forming cell assays. Hematopoietic progenitors overexpressing Cats (MIG-HA-Cats) formed considerably fewer GM (granulocytes and monocytes), BFU-E (erythrocytes) and GEMM (progenitors) colonies (60%, 76% and 67%, respectively) when compared to cells expressing MIG only (Figure 7A). Transduced cells expressing Cats showed a 45% decrease in the total number of colony forming units compared to control (MIG only) (Figure 7B) and formed 67% fewer secondary GM colonies than controls (Figure 7C).

## DISCUSSION

To explore the possibility that CATS might play a role in leukemogenesis, we performed an extensive analysis of CATS expression during induced differentiation of leukemia cell lines and functional characterization of CATS in the CALM/AF10-positive U937 leukemia cells.

Expression analysis on leukemic cell lines showed that CATS expression drastically decreased when the cells ceased to proliferate upon exposure to a variety of differentiation agents. Since we have previously shown that CATS is a marker for proliferation, the reduction of its expression during the cellular differentiations was expected.

However, differently from what was observed for erythroid, megakaryocytic and monocytic differentiation, granulocytic differentiation resulted in a significant increase in CATS expression. CATS upregulation was not dependent on the cell line, as it was observed for both U937 and NB4 ATRA-treated cells, but not for U937 PMA-treated cells (monocytic maturation). These results suggest that CATS may perhaps play a role in granulocytic differentiation or could be an ATRA responsive gene. Further studies will be necessary to address a possible role of CATS in granulocytic differentiation.

Using a lentivirus-mediated shRNA knockdown, we demonstrated that CATS silencing reduced cell proliferation and lowered the percentage of cells in the S-phase of the cell cycle. The inhibitory effect of CATS silencing on proliferation was discrete; nevertheless the data clearly demonstrates an altered control of cell cycle progression, attested by diminished expression of the cell cycle regulators CYCLIN A, CYCLIN E and CYCLIN B1, responsible for cell entry and progression in the S-phase and mitosis, respectively [34]. These results are consistent with the described accumulation of CATS protein in S, G_2_ and G_2_/M phases of the cell cycle and its suggested function in these phases [23, 25].

The reduced proliferation *in vitro* could not be documented *in vivo*, since growth of xenografts from U937 cells did not show any significant difference in tumor growth depending on the presence or absence of CATS. Therefore, CATS silencing was not sufficient to hinder tumor growth in this model.

In a previous study we identified KIS and SIVA1 as CATS interacting proteins ([24] and data not published). These proteins were recently reported to negatively regulate STATHMIN and consequently impair cell migration through altered microtubule dynamics [35, 36]. The interaction between CATS and the two regulators of STATHMIN prompted us to investigate whether CATS contribute to cellular migration. CATS silencing indeed resulted in less migration. Notably, STATHMIN and α-TUBULIN expression were downregulated in CATS silenced cells. The reduced protein expression was accompanied by lower levels of acetylated α-TUBULIN, a posttranslational modification only present on microtubule polymers (for review [37]) and phosphorylated Ser16 and Ser38 of STATHMIN, target residues for the CATS interacting proteins SIVA1 and KIS, respectively [24, 35, 36]. These results suggest that the reduced migratory ability of CATS depleted cells are due to interference with microtubule dynamics in these cells.

The connection of CATS with microtubule dynamics is very intriguing. Of note is the report that CATS functions during mitosis as a regulator metaphase-anaphase transition [25], a time when mitotic spindles move chromosomes.

Our data demonstrated that CATS depleted U937 cells form fewer colonies than control cells, leading to the conclusion that CATS may play a role in the clonogenicity of the U937 cell line. In an attempt to identify possible mechanism through which CATS could exert this function, we analyzed expression levels of self-renewal genes. We showed that *GLI-1*, a positive effector of the Hedgehog (Hh) signaling pathway [38], was significantly reduced in CATS depleted cells. Recently, Wellbrock and colleagues demonstrated *GLI1* expression as negative prognostic factor in AML. In that report, the authors showed that shRNA-mediated *GLI1* targeted inhibition ranged from 45% and 69% caused antileukemic effects, resulted in a significantly elevated induction of apoptosis and significantly reduced proliferation and colony formation in all three AML cell lines analyzed [39]. Thus, our findings indicate that the *GLI1* downregulation observed in CATS depleted U937 cells might be functionally relevant, and the impairment of this self-renewal pathway could be involved in the reduced colony forming ability of the CATS depleted cells.

Interestingly, *Cats* overexpression in murine primary bone marrow cells also lead to a decreased ability to form different types of colonies and reduced total cell number in colony forming cell assays. Taken together, these results suggest that any disturbance in the optimal expression level of CATS protein might have an impact on cell proliferation and clonogenicity.

Interestingly, in a very recent work using single cell RNA-Seq analysis of individual hematopoietic stem cells (HSCs) *Cats* (*Fam64a*) was found to be highly expressed in the HSC population primed for proliferation. Single-cell colony forming assays confirmed the high proliferation and differentiation potential of the *Cats*-expressing HSC population [40].

We have previously hypothesized that through its interaction with CALM CATS could play a role in CALM/AF10-mediated leukemogenesis [19]. Therefore, we analyzed the expression of the *HOXA* gene cluster, its co-factor *MEIS1* and *BMI1*, all known to be deregulated in CALM/AF10-positive leukemias [9, 13–15]. We observed a tendency of *HOXA5* upregulation in CATS depleted U937 cells compared to control cells. In CALM/AF10 leukemia, *HOXA5* overexpression results from hDOTL1-dependent retention of CALM/AF10 in the nucleus and local H3K79 hypermethylation at the *HOXA5* locus [15]. Just like hDOTL1, CATS is able of markedly increase the nuclear localization of the CALM/AF10 fusion protein [19]. Thus, it is tempting to speculate that CATS and hDOT1L compete for CALM/AF10 interaction, and upon CATS depletion, more hDOT1L is recruited to methylate the *HOXA5* locus. In addition to local H3K79 hypermethylation at the *HOXA5* locus, CALM/AF10-positive leukemia is characterized by a global reduction of the H3K79 epigenetic mark [16]. However, global H3K79 methylation did not change upon CATS depletion in U937 cells. Additional, more locus specific analysis of H3K79 methylation status after CATS depletion are now required.

Here we targeted CATS in a CALM/AF10 positive leukemia cell line and revealed some features of its function yet we should bear in mind the limitations of using this only cell line in the study. Therefore we can neither assume that our findings are restricted to U937 nor dependent on CALM/AF10. Although we have some *in vitro* evidences supporting the possible role of CATS in CALM/AF10-mediated leukemogenesis (demonstrated here and in previous publications [19, 23, 24]), the present study does not provide evidence for a role of CATS in *in vivo* CALM-AF10 leukemia.

Nevertheless our functional analysis demonstrates that CATS plays a role in cellular processes involved in tumorigenesis, such as cell cycle control, migration, clonogenicity and possibly self-renewal. Hence, further support the notion that disruption of CATS function might contribute to tumorigenesis.

## MATERIALS AND METHODS

### Leukemia cell lines

Leukemia cell lines U937, K562, NB4 and KU812, obtained from the American Type Culture Collection (ATCC, Manassas, USA) or the German Collection of Microorganisms and Cell Cultures (DSMZ, Braunschweig, Germany) were grown according to the suppliers' recommendations and used for cell differentiation, RNA and protein extraction, and virus transduction. All cell lines were tested and authenticated by STR matching analysis using the PowerPlex^®^ 16 HS system (Promega, Madison, WI, USA) and the ABI 3500 Sequence Detector System (Applied Biosystems, Foster City, CA, USA). Additionally, in U937 CALM/AF10 expression was confirmed by RT-PCR (data not shown).

### Differentiation of cell lines

RNA and protein samples of KU812, K562 and NB4 leukemia cells induced to differentiate were obtained from previous study [41] and *CATS* expression was assessed in the Hemin and Hydroxyurea (HE-HU)-induced erythroid differentiation of KU812, the phorbol-13 myristate-12 acetate (PMA)-induced megakaryocytic differentiation of K562 and the all-trans retinoic acid (ATRA)-induced granulocytic differentiation of NB4 cells.

U937 cells were induced to differentiate for 2 and 4 days into monocytes and granulocytes with PMA (20 nM) and ATRA (10^−6^ M) treatment, respectively. Differentiation was monitored by flow cytometry for positive staining of the cell surface markers CD11b, CD14 and CD15 and by qPCR for transcript levels of *CD15* and *GCSFR*. Cells were collected for morphology analysis and for RNA and protein extraction (Supplementary Figure S1).

### Quantitative PCR

Total RNA was isolated using the RNeasy^®^ Mini or Micro Kit (Qiagen, Hilden, Germany). DNAse I treated RNA was reverse transcribed with oligo dT primers and RevertAid™ First Strand cDNA Synthesis Kit (MBI Fermentas, St. Leon-Rot, Germany). Reactions were carried out with Maxima SYBR Green qPCR master mix, according to the manufacture's protocol (MBI Fermentas, St. Leon-Rot, Germany). The plates were run and analyzed by Mastercycler^®^ ep realplex 4 System (Eppendorf, Hamburg, Germany). Primer sequences are available upon request. *HPRT* and *GAPDH* were used as endogenous control and relative gene expression was calculated using the 2^−ΔΔC^_T_ equation [42].

### Immunobloting

Cellular lysates were electrophoresed on 10-12% SDS-PAGE and transferred to nitrocellulose membrane (Hybond™ ECL™, GE Healthcare, Buckinghamshire, UK). The membranes were blocked and probed with specific antibody, followed by detection with fluorescently labeled secondary antibodies. Membranes were visualized on Alliance 2.7 (UVItec, Cambridge, England). Primary antibodies were purchased from Santa Cruz Biotechnologies, unless otherwise specified: anti-ACTIN (I-19; sc-1616) (1:2000), anti-GAPDH (6C5; sc-32233) (1:4000), anti-CYCLIN E (E-4; sc-377100) (1:1000), anti-CYCLIN B1 (D-11; sc-7393) (1:1000), anti-CYCLIN D1 (A-12; sc-8396) (1:1000), anti-CYCLIN A (H-3; sc-271645) (1:2000), anti-α-TUBULIN (B-7; sc-5286) (1:2000), anti-SIVA (C-20; sc-7436) (1:1000), anti-pOP18 S16R (sc-12948) (1:2000), anti-pOP18 S38 (sc-101810) (1:1000), anti-OP18 (E-3; sc-55531) (1:2000), anti-H3 (C-16; sc-8654) (1:1000). Anti-H3K79 (ab3594) (1:1000) and anti-ACETYL-α-TUBULIN (6-11B-1; ab24610) (1:2000) were purchased from Abcam, Cambridge, England and anti-CATS 2C4 (1:250) was previously described [23]. Secondary antibodies were purchased from Life Technologies (Carlsbad, CA, USA): anti-mouse (1:5000), anti-rat (1:2000), anti-rabbit (1:10000) and anti-goat (1:4000). Quantitative analyses of the optical intensities protein bands were determined with UN-SCAN-IT gel 6.1 software (Silk Scientific, Inc., USA) and normalized by control protein.

### Transduction of U937 cells

U937 cells (American Type Culture Collection (ATCC, Manassas, U.S.A) were transduced with lentivirus particles expressing a pool of two short hairpin RNAs (shRNA) targeting the CATS sequence (FAM64A sc-93656-V; Santa Cruz Biotechnologies, CA, USA; siRNA target sequences GCUUCAUACUCAAGGAUGUtt and GAAGUGCUAGCAUCAGAUAtt) or nonspecific control target (sc-108080), named shCATS and shControl cells, respectively. Briefly, 2 × 10^5^ cells were transduced by spinoculation at multiplicity of infection (MOI) equal to 1 and stable polyclonal shCATS and shControl cell lines were generated after 15 days selection with puromycin (10 μg/ml).

### Proliferation assays

For MTT assay cells were seeded in 96-well plates at density of 2.5 × 10^4^ cells/well. After 24 or 48 hours of incubation under normal culture condition, 10 μl of a 5 mg/ml solution of MTT was added per well and incubated for 4 hours. Reaction was stopped by adding 100 μl of 0.1N HCl/isopropanol and proliferation evaluated by measuring the absorbance at 570 nm with an automated plate reader.

Cell proliferation was further assessed by Ki-67 staining. ShCATS and control cells were stained with Ki-67 antibody according to manufacturer's instructions (Ki-67 PerCP-Cy5.5 clone B56; BD Bioscience, San Jose, CA, USA). The mean of fluorescence intensity (M.F.I) was obtained by FACS analysis using a FACSCalibur (Becton Dickinson, San Jose, CA, USA) and FlowJo software. IgG isotype was used as negative control for each condition and ten thousand events were acquired for each sample.

### Cell cycle analysis

2.5 × 10^5^ cells were fixed in 70% ethanol for 30 min on ice, washed with PBS and stained with 20 μg/ml propidium iodide (PI) containing 10 μg/ml RNAse A for 30 min at room temperature (RT). Cell cycle analysis was performed using FACSCalibur (Becton-Dickinson, California, USA) and Modfit (Verify Software House Inc., USA).

### Apoptosis assays

For apoptosis evaluation, cells seeded in 12-well plates were treated with UV irradiation (40 J/m^2^) and collected at 0, 3, 6, 9 and 12 hours after UV exposure. At the indicated time points, cells were washed with ice-cold PBS and stained with annexin V and PI (BD Biosciences Pharmingen, California, USA) for 15 minutes at RT. Apoptosis analysis was performed using FACSCalibur (Becton-Dickinson) and FACSDiva software (BD Biosciences Pharmingen). Ten thousand events were acquired for each sample.

### Xenograft model of tumor growth in NOD/SCID mice

For the xenograft tumor model, 1 × 10^7^ U937 shCATS or shControl transduced cells were implanted into the dorsal sub cutis of 8-12 week-old female non-obese diabetic/severe combined immunodeficiency (NOD/SCID) mice. Tumors grew locally for 12 days, then they were excised, measured and weighted. Tumor measurements were converted to tumor volume (V) by the formula (V = W^2^ x L x 0.52), where W and L stands for smaller and larger diameters, respectively. RNA was extracted from tumors samples.

### Migration assay

Migration assays were performed in 12-well 8μm pore-sized transwell plates (Costar, Corning, NY, USA). Cells were seeded above the filters at a density of 1 × 10^5^ cells/well. The lower compartment was filled with the following media: 10% FBS/0.5% BSA; 200 ng/mL CXCL12/0.5% BSA (PeproTech, Rocky Hill, NJ, USA); 10% FBS/200 ng/mL CXCL12/0.5% BSA and 0.5% BSA was used as negative control. After 24 hours, the number of cells which migrated through the filter and reached the lower compartment was counted. Values were expressed as percentage of the input (cells applied directly to the lower compartment) set as 100%.

### Colony forming assay

shControl and shCATS transduced U937 cells were plated in semisolid medium depleted of any growth factors (5 × 10^2^ cell/ml; MethoCult 4230; StemCell Technologies Inc., Vancouver, Canada). Colonies were detected after 8 days of culture by adding 200 μl of a 5 mg/ml MTT solution and scored by Image J quantification software (NIH, Bethesda, Maryland, USA).

### Construction of retroviral vector and virus production

Murine *Cats* hemagglutinin (HA)-epitope tagged cDNA was generated by PCR and subcloned into the multiple cloning site of the modified murine stem cell virus vector (MIG), upstream of the internal ribosomal entry site (IRES) and enhanced GFP (EGFP) gene (MIG-HA-Cats). The MIG vector carrying only the IRES-EGFP cassette was used as a control.

Production of high-titer helper-free recombinant retrovirus was carried out following standard procedures [43] by using the ecotropic 293-Phoenix cell line and the packing cell line GP^+^E86 [44]. Briefly, 293-Phoenix cells were first transfected with the MIG or MIG-HA-Cats plasmids. Supernatant was used to infect the GP^+^E86 cells, which were sorted based on GFP expression. Single cell culture was carried out in order to obtain monoclonal populations of GP^+^E86-MIG or MIG-HA-Cats viral producer cells. The viral titre was determined by transducing NIH3T3 cells.

### Retroviral transduction of primary bone marrow cells

Primary bone marrow cells were obtained from 8 to 12-week-old C57BL/6 mice pretreated with 5-fluorouracil for 5 days. Total bone marrow was harvested from femurs and cultured in DMEM, 20% FBS (Gibco by Life Technologies, Carlsbad, CA, USA) and cytokines (100 ng/mL stem cell factor (SCF), 10 ng/mL interleukin 6 (IL6) and 6 ng/ml interleukin 3 (IL3)) for 48 hours. For retrovirus transduction, 5 × 10^6^ cells were cocultured with 1.2 × 10^6^ virus-producing GP^+^E86 cells (GP^+^E86-MIG and -MIG-HA-Cats) in the presence of cytokines and protamine sulfate (5 μg/mL) (Sigma-Aldrich, St. Louis, MO, USA). The transduction was stopped by removing the bone marrow cells from the GP^+^E86 cells and culturing them in afore mentioned media for another 48 hours to allow for GFP expression. Retrovirally transduced cells were sorted based on GFP expression on a FACSAria IIu (Becton-Dickinson). Sorted GFP-positive cells were further cultured for 48 hours in DMEM, 35% FBS plus cytokines and used for colony forming cell (CFC) assays and RNA extraction.

### Colony forming cell assay

2 × 10^4^ transduced cells were cultured in 1% myeloid-conditioned methylcellulose medium (MethoCult M3434; StemCell Technologies Inc., Vancouver, Canada) for 8 days, after which GM (granulocytes and monocytes), BFU-E (erythrocytes) and GEMM (progenitors) colonies were counted. The total number of cells was determined; cells were replated in the same concentration and cultured for an additional 10 days, after which secondary colonies were counted.

### Animal care

Animal care was conducted in accordance with the standard ethical guidelines. Protocols have been approved by the local ethical committee “Comissão de Ética no Uso de Animais (CEUA)/Unicamp” - Protocol n° 3165-1. Parental strain mice were bred and maintained at the University of Campinas Central Breeding Center (Campinas, SP, Brazil).

## SUPPLEMENTARY MATERIALS FIGURES


