# Supplementary material for: CATS (FAM64A) abnormal expression reduces clonogenicity of hematopoietic cells
Source: Oncotarget. 2016 Aug 31;7(42):68385–96. doi: 10.18632/oncotarget.11724 (PMC5356563; doi:10.18632/oncotarget.11724)
Supplement: Supplementary file 1 [file oncotarget-07-68385-s001.pdf]

CATS (FAM64A) abnormal expression reduces clonogenicity of hematopoietic cells

SUPPLEMENTARY FIGURES

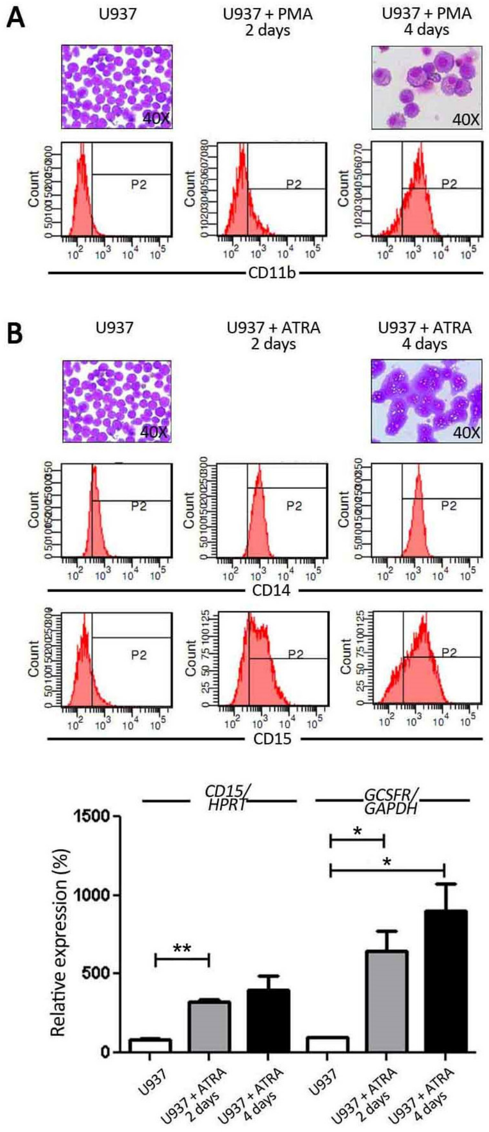

Supplementary Figure S1: U937 differentiation into monocytes and granulocytes. A. Rosenfeld-stained cytospin preparation (upper panels) and expression of the CD11b cell surface marker (lower panel) of undifferentiated cells (U937) and cells differentiated into monocytes after 2 and 4 days of treatment with PMA. **B.** Rosenfeld-stained cytospin preparation (upper panels), expression of cell the surface markers CD14 and CD15 assessed by flow cytometry (middle panels) and relative expression of *CD15* and *GCSFR* of undifferentiated cells (U937) and cells differentiated into granulocytes after 2 and 4 days of treatment with ATRA. Expression levels of *CD15* mRNA were normalized by *HPRT* and expression levels of *GCSFR* mRNA were normalized by *GAPDH*. *p<0.05; **p<0.01, Student's t test.

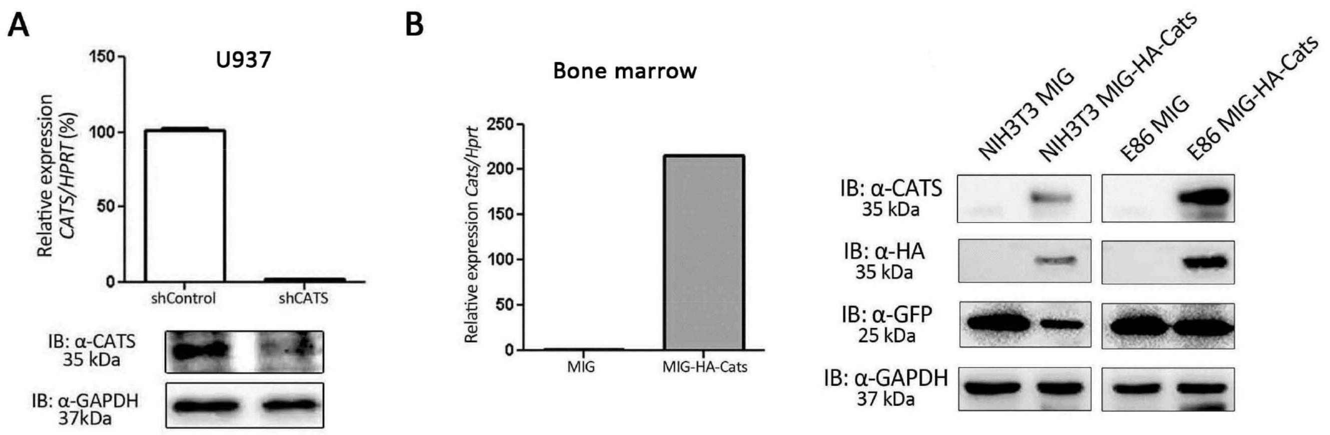

Supplementary Figure S2: CATS depletion and overexpression was confirmed. A. Relative expression of *CATS* in control (shControl) and CATS depleted U937 cells (shCATS). Expression levels of *CATS* mRNA were normalized by *HPRT*. Western blot analysis of shControl and shCATS total cell extracts (upper panel). Membrane was blotted against anti-CATS 2C4 and anti-GAPDH, used as a loading control (lower panel). **B.** (left panel) Relative expression of *Cats* in the MIG and MIG-HA-Cats transduced primary bone marrow cells. Expression levels of *Cats* mRNA were normalized by *Hprt*. (right panel) Western blot analysis on total cell extracts of NIH3T3 and E86 cells transduced either with MIG or MIG-HA-Cats. Membrane was blotted against anti-CATS 2C4, anti-HA, anti-GFP and anti-GAPDH, used as a loading control.

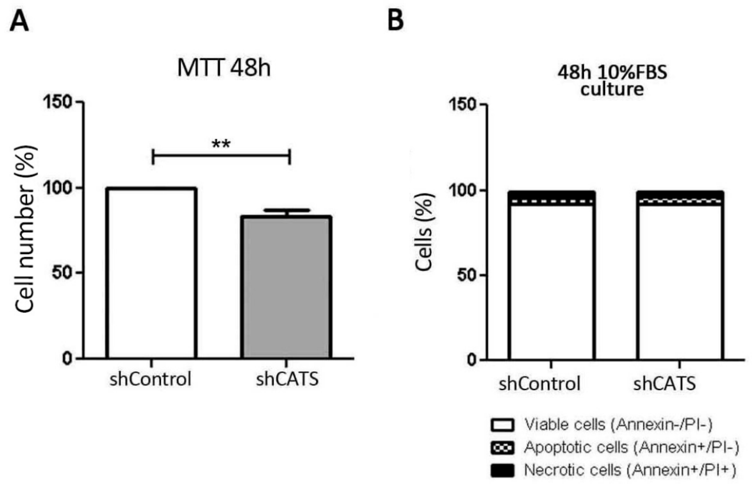

Supplementary Figure S3: MTT and apoptosis analysis. A. Cell proliferation was determined by MTT assay after 48h of normal culture conditions. Results are shown as mean ± standard deviation (SD) of five independent sixplicates experiments. **B.** Apoptosis was determined by staining cells with Annexin V and PI, followed by flow cytometry analysis. Results are shown as mean ± SD of four independent experiments. **p<0.01, Student's t test.

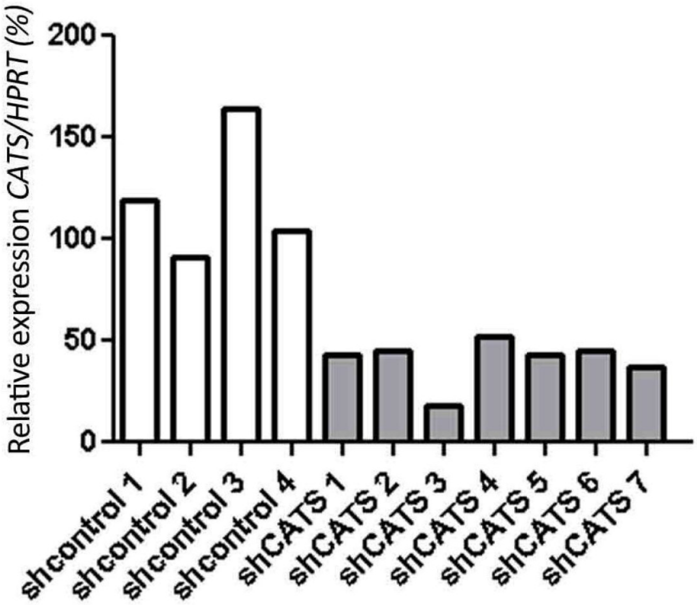

Supplementary Figure S4: CATS depletion was confirmed in the excised tumors samples. Relative expression of *CATS*. Expression levels of mRNA were normalized by *HPRT*.

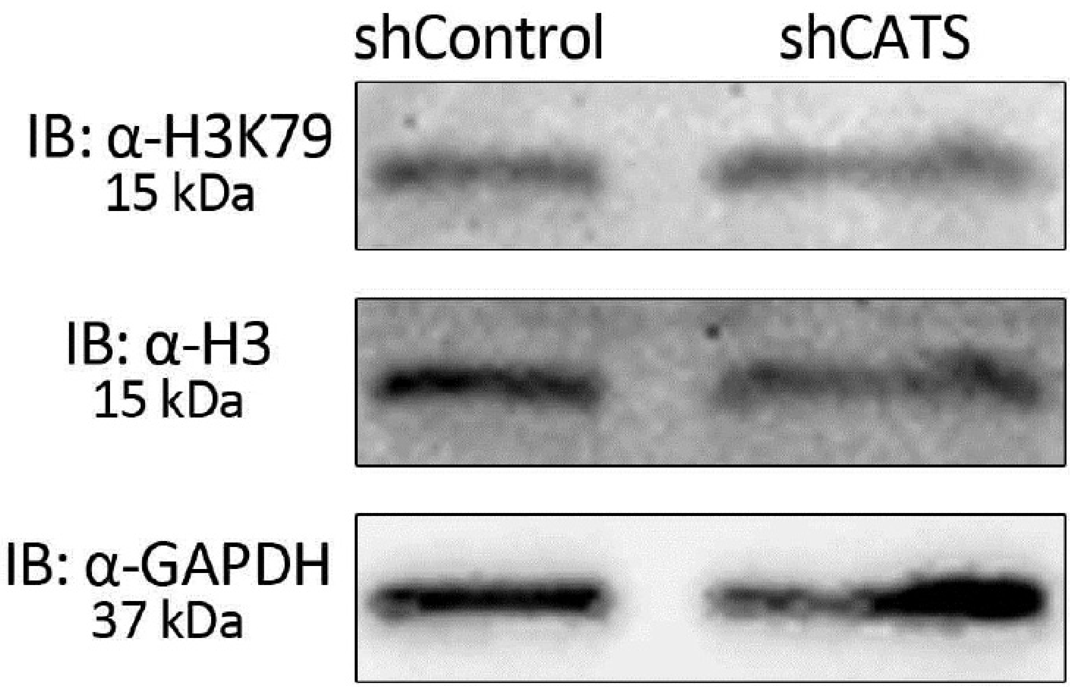

Supplementary Figure S5: Global methylation of H3K79 in CATS depleted U937 cells. Western blot analysis of shControl and shCATS total cell extracts. Membrane was blotted against anti-H3K79, anti-H3 and anti-GAPDH, as a control for equal sample loading.
